# Functional genomics and small molecules in mitochondrial neurodevelopmental disorders

**DOI:** 10.1016/j.neurot.2024.e00316

**Published:** 2024-01-19

**Authors:** Daniel G. Calame, Lisa T. Emrick

**Affiliations:** a Section of Pediatric Neurology and Developmental Neuroscience, Department of Pediatrics, Baylor College of Medicine, Houston, TX, USA; b Texas Children's Hospital, Houston, TX, USA; c Department of Molecular and Human Genetics, Baylor College of Medicine, Houston, TX, USA

**Keywords:** Mitochondrial disease, Functional genomics, Neurodevelopmental disorders, Small molecules, Therapeutics

## Abstract

Mitochondria are critical for brain development and homeostasis. Therefore, pathogenic variation in the mitochondrial or nuclear genome which disrupts mitochondrial function frequently results in developmental disorders and neurodegeneration at the organismal level. Large-scale application of genome-wide technologies to individuals with mitochondrial diseases has dramatically accelerated identification of mitochondrial disease-gene associations in humans. Multi-omic and high-throughput studies involving transcriptomics, proteomics, metabolomics, and saturation genome editing are providing deeper insights into the functional consequence of mitochondrial genomic variation. Integration of deep phenotypic and genomic data through allelic series continues to uncover novel mitochondrial functions and permit mitochondrial gene function dissection on an unprecedented scale. Finally, mitochondrial disease-gene associations illuminate disease mechanisms and thereby direct therapeutic strategies involving small molecules and RNA-DNA therapeutics. This review summarizes progress in functional genomics and small molecule therapeutics in mitochondrial neurodevelopmental disorders.

## Introduction

Human neurodevelopment occurs in an orderly fashion from neurulation onto adulthood. Patterns of developmental attainment are well-characterized and include clinical (*e.g.*, motor, language, social, cognitive) and biological (*e.g.*, anatomic, cellular, network, biochemical) milestones. The precise timing and order of milestone attainment varies from individual-to-individual; significant delays or disordered patterns of milestone attainment are the defining feature of neurodevelopmental disorders. The precise definition of neurodevelopmental disorders varies. The most expansive definitions encompass 11–23.9 ​% of the population and include common disorders like attention deficient hyperactivity disorder (ADHD), dyslexia, and learning disabilities [1]. The spectrum of neurodevelopmental disorders ranges from common conditions to rare disorders with more substantial impacts on motor, behavioral, and cognitive function like autism, intellectual disability (ID), and cerebral palsy. While neurodevelopmental disorders may result from environmental factors or acquired brain injuries, genetic factors play an important role across the entire developmental disorder spectrum. Human genetics has demonstrated common disorders like ADHD are primarily complex or polygenic traits [2], whereas rare, severe neurodevelopmental disorders like intellectual disability are often monogenic or Mendelian traits [3]. Mendelian neurodevelopmental disorders are highly heterogeneous [4,5]. Their discovery rate has dramatically accelerated with advances in genomics; this is attested by the near doubling of ID-associated genes in a seven-year span (2016–2023) from approximately 800 to over 1500 [5]. These discoveries provide answers for patients and families with neurodevelopmental disorders and frequently conclude a long ‘diagnostic odyssey.’ They also illuminate the complex biological pathways underlying human brain development.

**Fig. 1:**
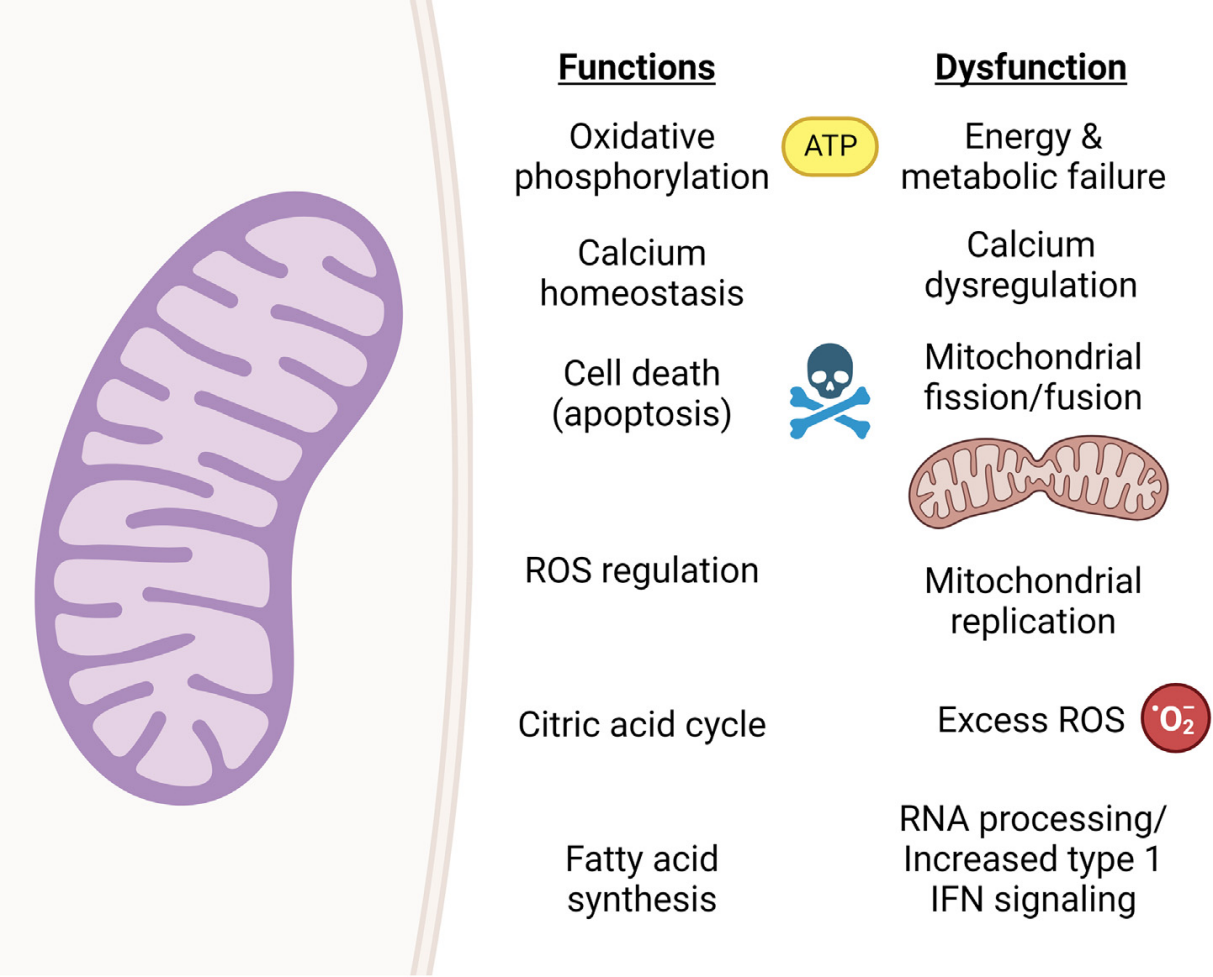
**Physiological functionsof mitochondria and examples of mitochondrial dysfunction in neurodevelopmental disorders.** Physiologic functions of mitochondria are shown in the left column. Examples of mitochondrial dysfunction responsible for mitochondrial neurodevelopmental disorders are shown in the right column. Figure created using Biorender.com.

Mitochondrial dysfunction is often implicated as a common pathway in neurodevelopmental disorders [6,7]. Mitochondria are the “powerhouse of the cell” which generate adenosine triphosphate (ATP) through aerobic respiration. As only liver and muscle exceed the brain's energy demands, any failure of mitochondrial energy production can have disastrous neurological consequences [8]. Mitochondrial energy production is particularly important during the neurodevelopmental transition from glycolysis-dependent neuronal stem cells to oxidative phosphorylation (OXPHOS)-dependent neurons [6]. Mitochondria also have essential functions outside energy production including calcium buffering (Fig. 1). They are dynamic organelles migrating throughout neurons and undergoing continuous cycles of fusion and fission to replenish mitochondrial pools [7]. Consequently, pathogenic variation in genes with an integral role in mitochondrial migration, fission and fusion like *MSTO1*, *MFN2*, *OPA1*, *YME1L1*, *DNM1L*, *MFF* and *FBXL4* often manifests clinically in neurodevelopmental disabilities [9,10].

Each mitochondrion contain its own 16.5 kilobase genome (mtDNA) with 37 genes encoding 13 proteins, 22 transfer RNAs (tRNA), and 2 ribosomal RNAs (rRNA) [11,12]. Unlike the nuclear genome, mtDNA is exclusively inherited from an individual's mother. Each cell contains multiple mitochondria and therefore multiple copies of the mitochondrial genome allowing for homoplasmy and heteroplasmy. Homoplasmy refers to a state of uniformity in which all copies of mtDNA contain a particular genetic variation. Heteroplasmy occurs when only a fraction of the multiple copies of mtDNA contains a particular genetic variation. The degree of heteroplasmy in separate tissues can increase or decrease throughout life depending on selective pressure; consequently, mitochondrial dysfunction may worsen or improve throughout the individual lifespan. RBCs have a short life cycle, and therefore mtDNA variation heteroplasmy levels may decrease over time secondary to mtDNA turnover and lead to difficulty with detection and diagnosis based on blood alone. Thus, muscle or liver testing may be required to detect heteroplasmic mtDNA variations. Maternal mtDNA inheritance, heteroplasmy, and homoplasmy also result in mitochondrial disease intrafamilial variability. An asymptomatic mother with low level heteroplasmy for a pathogenic mtDNA variant may have children with highly variable disease severity depending on each child's degree of heteroplasmy. While mtDNA is essential for mitochondrial function, mtDNA alone is insufficient for mitochondrial homeostasis. As the mitochondrial proteome is estimated to contain over 1500 proteins while the mitochondrial genome contains only 37 genes, most mitochondrial proteins are encoded by the nuclear genome [13,14]. These nuclear encoded mitochondrial genes are often essential for mitochondrial function and typical neurodevelopment; consequently, individuals with mitochondrial neurodevelopmental disorders are more likely to have pathogenic variation in nuclear encoded genes than genes within the mitochondrial genome.

## Functional genomics of mitochondrial neurodevelopmental disorders

### Traditional approaches and cellular models of mitochondrial neurodevelopmental disorders

There are a multitude of well-established laboratory and imaging biomarkers of mitochondrial neurodevelopmental disorders including elevated plasma and cerebrospinal fluid lactate levels, organic aciduria, low plasma carnitine, elevated alanine, deep gray matter signal hyperintensity on magnetic resonance imaging (MRI), and lactate peak of magnetic resonance spectroscopy (MRS) [15]. Serum levels of growth factors GDF15 and FGF21 are elevated in some individuals with mitochondrial disorders but can be elevated in other clinical settings such as inflammation and acute illness [[16], [17], [18]]. Other more sensitive and specific assays include quantitative assessment of OXPHOS enzyme activity, blue-native electrophoresis (BN-PAGE), mitochondrial copy number in tissue for DNA depletion, and gene-specific assays [12,15].

Cellular models to characterize the functional impact of mitochondrial genetic variation *ex vivo* include 1) cybrids, cell lines generated from the fusion of patient-derived mitochondria with a cell line lacking functional mitochondria; 2) patient-derived primary cells or immortalized cell lines including skin fibroblasts and lymphoblastoid cell lines; 3) patient-derived induced pluripotent stem cells; and 4) patient-derived induced neurons. Animal models including rodents, pigs, zebrafish, roundworms, and fruit flies have also been extensively used to characterize the impact of mitochondrial gene deletion or even human genetic variation through genetic variant knock-in at the organismal level [19,20].

Induced pluripotent stem cells (iPSCs) are produced by reprogramming primary cells like fibroblasts and peripheral blood mononuclear cells (PBMCs) into an embryonic stem cell-like state via the Yamanaka factors [21]. iPSCs maintain pluripotency in culture and can be expanded and differentiated into many cell types within the developing brain including neuronal progenitor cells (NPC), neurons, astrocytes, oligodendroglial cells, and microglia. Abnormalities identified in genetic neurodevelopmental disorders have been recapitulated in iPSC-derived NPCs and neurons from patients with neurodevelopmental disorders as well as iPSCs genetically engineered to contain pathogenic variants [22]. Thus, iPSC-derived neurons are a well-established human cellular model for the study of mitochondrial neurodevelopmental disorders with the potential to be used in high-throughput functional genomics studies.

The production, maintenance, and differentiation of iPSCs is costly, technically challenging, and time-consuming. An alternative *ex vivo* model of human neurons with the potential to be less costly and time-consuming than iPSCs are inducible neurons (iNeurons) [23,24]. iNeurons are produced via the induction of neuronal transcription factors in fibroblasts or PBMCs. They closely resemble primary neurons morphologically and express neuronal markers. They also fire action potentials and form mature synapses. To date, iNeurons have not received much study in neurodevelopmental disorders but have shown promise in the study of neurodegenerative diseases.

### Genomic sequencing

**Fig. 2:**
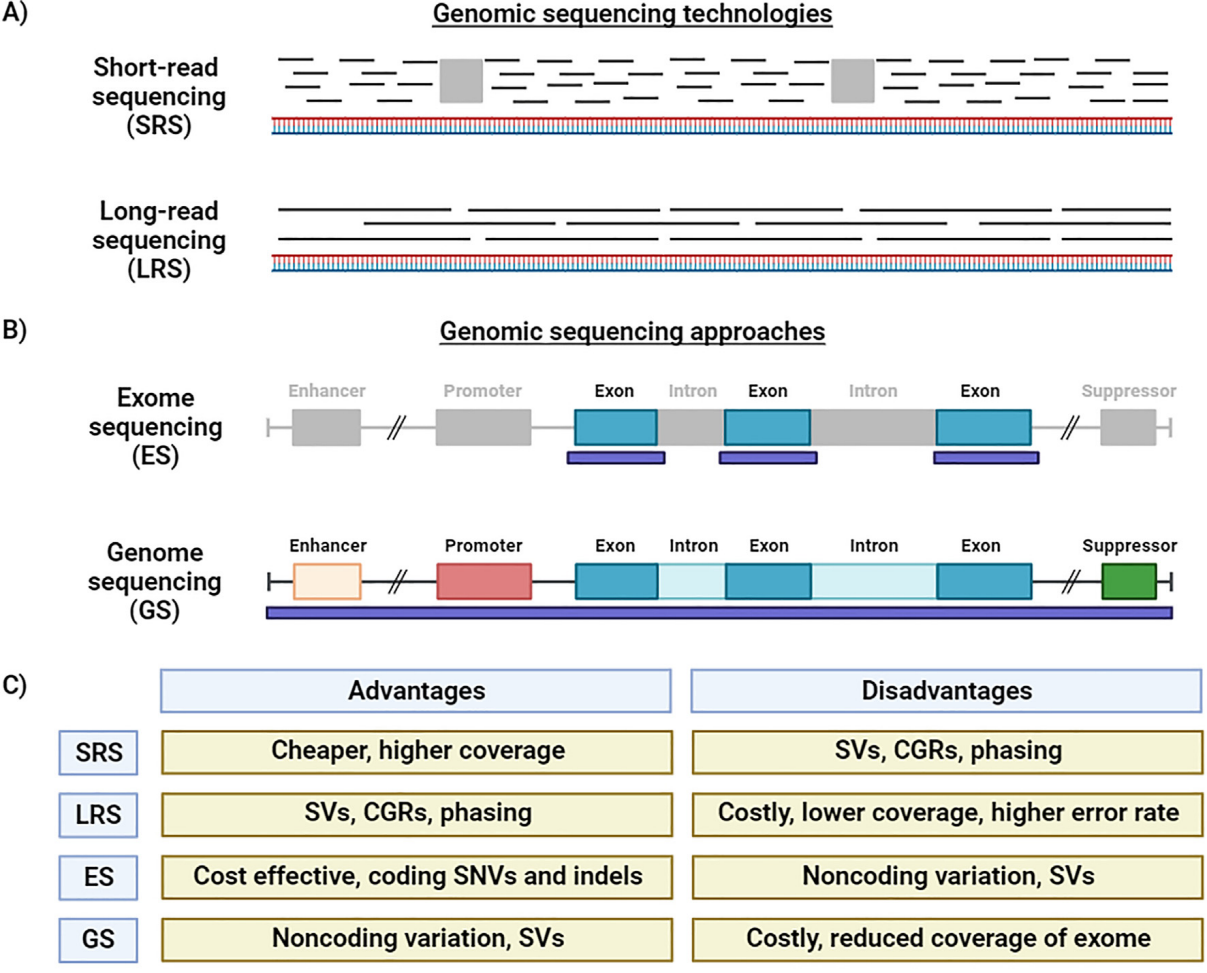
**Genomic approaches to mitochondrial neurodevelopmental disorders.** A) Short-read sequencing (SRS, also known as next generation sequencing, NGS) is the most common technology used in genomic sequencing. In SRS, sequencing data consists of small (50–150 bp) sequences called reads. Short-read data must be computationally assembled to generate genomic sequences. Gaps in genome coverage (*gray rectangles*) in SRS can limit resolution of complex genomic regions (CGR) and structural variants (SV). In contrast, long-read sequencing technologies generate kilobase to megabase sequencing reads. Long sequencing reads provide better coverage without gaps across the genome. They improve SV and CGR resolution and played an integral role in the Telomere-to-Telomere (T2T) project. B) Exome sequencing (ES) involves the selective capture and sequencing of the exome. The exome is composed of all protein-coding genes and makes up ∼1 ​% of the genome. Capture regions (*purple rectangles*) often include flanking intronic regions since intronic genetic variants near splicing junctions often cause splicing dysfunction. Genome sequencing (GS) involves sequencing of the entire genome including introns, promoters, and intergenic regions including regulatory elements like enhancers and suppressors. C) The advantages and disadvantages of SRS, LRS, ES, and GS are shown. SVs ​= ​structural variants; CGRs ​= ​complex genomic regions; SNVs ​= ​single nucleotide variants; indels ​= ​small insertion and deletions. Figure created using Biorender.com.

The cornerstone of functional genomics is genomic sequencing (Fig. 2). Advances in genomic technologies over the past twenty years have enabled high-throughput genomic investigations in cellular models, model organisms, primary cells, and biospecimens. Short-read sequencing (SRS), also known as large-scale massively parallel sequencing or next generation sequencing (NGS), has transformed genomics from its introduction (Fig. 2A) [25,26]. SRS generates a tremendous volume of short (∼50–150 base pairs, bp) DNA sequence fragments known as reads. Each nucleotide is sequenced through multiple reads; genomic sequencing may involve anywhere from 10 to 100s of reads per nucleotide with ultra-high read depths used to study somatic mosaicism. Each read must be computationally mapped to the reference genome and assembled to generate gene or genome-level sequence. At present, SRS is the most widely used genomic sequencing approach. With adequate read depth, SRS has excellent sensitivity for detection of single nucleotide variants (SNVs), indels, some structural variants, somatic mosaicism, and tandem repeat expansions [[27], [28], [29], [30], [31]].

Another major milestone in genomics was the development of capture methods allowing for targeted sequencing of genomic regions of interest by SRS [32,33]. Capture methods enabled exome sequencing (ES), the selective sequencing of all exons within the genome (Fig. 2B). Exons are the protein-coding portions of genes which are transcribed as messenger RNA (mRNA). The exome makes up approximately 1 ​% of the whole genome. ES is highly sensitive for the detection of coding SNVs and indels. It can also detect large copy number variants (deletions and duplications) and unbalanced translocations. ES has proven highly efficient at identifying pathogenic coding variation in mitochondrial disease genes encoded within the nuclear genome.

In recent years, genome sequencing (GS) has been increasingly utilized in research and clinical settings (Fig. 2B). GS encompasses the entire nuclear genome as well as the mitochondrial genome. Consequently, GS is more costly but more comprehensive than ES. GS is superior to ES for the detection of structural variants; it also detects noncoding variants in promoters, deep intronic regions, and other regulatory elements like enhancers and suppressors. Noncoding variants are increasingly recognized as a cause of Mendelian neurodevelopmental disorders [34,35].

Another recent advance is long-read sequencing technologies (LRS) like single-molecule real-time (SMRT) and Nanopore sequencing. LRS can produce sequence reads in excess of 10 kilobases [27,36]. The major advantage of LRS over SRS is a significant reduction in sequencing gaps (Fig. 2A–C). This improves read mapping and thereby helps resolve repetitive complex genomic regions (CGRs) like *Alu* elements, long interspersed nuclear elements (LINE), segmental duplications, and centromeres. It also improves the precise resolution of structural variants. LRS can also determine the phase of variants at a single locus if both variants are close enough to permit their capture within single reads [37]. LRS played an integral role in the Telomere-to-Telomere (T2T) project generating the first complete haploid genome assembly [38] and the human pangenome reference, a collection of 47 phased, diploid genome assemblies from a genetically diverse cohort [39]. In addition, LRS platforms can simultaneously profile chromatin accessibility and methylation states. The major disadvantages of LRS at present are high cost, requirement for high molecular weight DNA to maximize sequencing read length, and higher error rate (Fig. 2C). Technical advances like circular consensus SMRT sequencing have improved error rates and accuracy of LRS [40]. The application of LRS to rare Mendelian mitochondrial neurodevelopmental disorders is ongoing [41,42]. Bulk genomic sequencing of tissues, biospecimens, or cell cultures should also be distinguished from single cell genomic sequencing. The recent application of single cell genomic sequencing to mitochondrial disorders and has demonstrated distinctive patterns of purifying selection across blood and immune cell lineages [43,44].

The interpretation of genetic variation has been facilitated by the development of genomic databases. Since mitochondrial neurodevelopmental disorders are rare because of negative selection due to their severe phenotypic consequences, their underlying pathogenic genetic variants are generally rare (allele frequencies <1 ​%). Common genetic variants (allele frequencies >1 ​%) within mitochondrial disease genes can be concluded to represent benign polymorphisms using the same logic. Therefore, estimations of variant allele frequencies within human populations are essential for variant interpretation. One major resource of variant allele frequencies is the Genome Aggregation Database (gnomAD) [45,46]. gnomAD was launched in 2014 to aggregate exome and genome sequencing data for the clinical and research human genetics communities. The latest version v4.0.0 contains ES data from 730,947 individuals and GS data from 76,215 individuals. The dataset can be downloaded and used for variant annotation and filtering; it is also accessible via a user-friendly web portal to quickly assess the allele frequency of any variant of interest. Pathogenic *de novo* variants in individuals with sporadic severe neurodevelopmental disorders should be absent or extremely rare in population databases; higher allele frequencies are incompatible with a severe *de novo* disease model. Allele frequencies of pathogenic variants in genes associated with severe recessive diseases can be higher than those associated with severe dominant disorders, but they should not be present in the homozygous state. As some genetic variants can be extremely rare in one population but more common in others, the lack of diversity in genomic datasets is an important limitation which must be considered [47]. In addition to allele frequencies, gnomAD also contains helpful gene-level metrics including constraint against missense and predicted loss-of-function (pLoF) variation and regional missense variation constraint. Genes with the highest degree of pLoF constraint (probability of being loss-of-function intolerant [pLI] >0.9) are enriched for haploinsufficiency. Haploinsufficiency refers a requirement for two functional copies of a gene for normal health: loss of one copy of a haploinsufficient gene due to a loss-of-function allele results in a disease phenotype. High levels of regional missense variation constraint correspond to critical functional domains like helicase domains and protein binding sites. Constraint metrics can help prioritize and interpret genetic variation in known and novel disease genes. Genetic variants in highly constrained genes or gene regions are anticipated to be more deleterious than genetic variants in less constrained genes or gene regions.

Other useful genomic databases include ClinVar, Human Gene Mutation Database (HGMD), Leiden Open Variation Database (LOVD), and MITOMAP [[48], [49], [50], [51]]. These databases aggregate human genetic variation reported in the medical and scientific literature and genetic variation identified in clinical diagnostic laboratories. Gene variant entries in ClinVar, HGMD, and LOVD include clinical classifications – pathogenic, likely pathogenic, variant of uncertain significance, likely benign, and benign – according to American College of Medical Genetics and Genomics criteria [52]. The MITOMAP database specifically compiles genetic variation in the mitochondrial genome and thus can be useful to discriminate between benign mitochondrial polymorphisms and pathogenic genetic variants. Thus, genomic databases like gnomAD, ClinVar, and MITOMAP can be quickly queried for any variant to determine allele frequency and clinical significance if known.

*In silico* software prediction programs also play an important role in human genetic variant interpretation and functional genomics. These tools leverage evolutionary conservation, variant allele frequencies, and protein structure/function to predict a genetic variant's impact on gene function. Ensemble methods like CADD and REVEL incorporating multiple *in silico* tools and/or trained on known pathogenic and benign genetic variants have demonstrate superior performance over individual tools [53,54]. More recently, programs like Alpha Missense, ESM1b, PrimateAI-3D, EVE, MAVERICK, SpliceAI and Pangolin have utilized unsupervised deep learning models to provide genome-wide predictions of variant effect [[55], [56], [57], [58], [59], [60], [61]]. These models consistently outperform older *in silico* predictive models.

### Transcriptomics

Despite advances in our understanding of regulatory elements, transcription, and RNA splicing, it remains difficult to predict the functional consequence of genetic variation within noncoding regions of the genome. This is problematic as pathogenic noncoding variation can alter gene expression or RNA splicing and is increasingly found to underlie Mendelian neurodevelopmental disorders [34,62]. For example, genetic variants within promoters, enhancers, or long noncoding RNAs may increase or decrease gene expression and thereby cause disease. Similarly, genetic variation in deep intronic regions may disrupt intronic splicing enhancers and silencers and therefore cause aberrant inclusion of introns or exon skipping. While deep learning models like SpliceAI and Pangolin have improved predictions of splicing abnormalities resulting from coding and non-coding single nucleotide variants and indels [59,60,63], these *in silico* predictive models continue to have significant limitations demonstrated by numerous examples of pathogenic splicing variants not predicted by these programs [64,65].

**Fig. 3:**
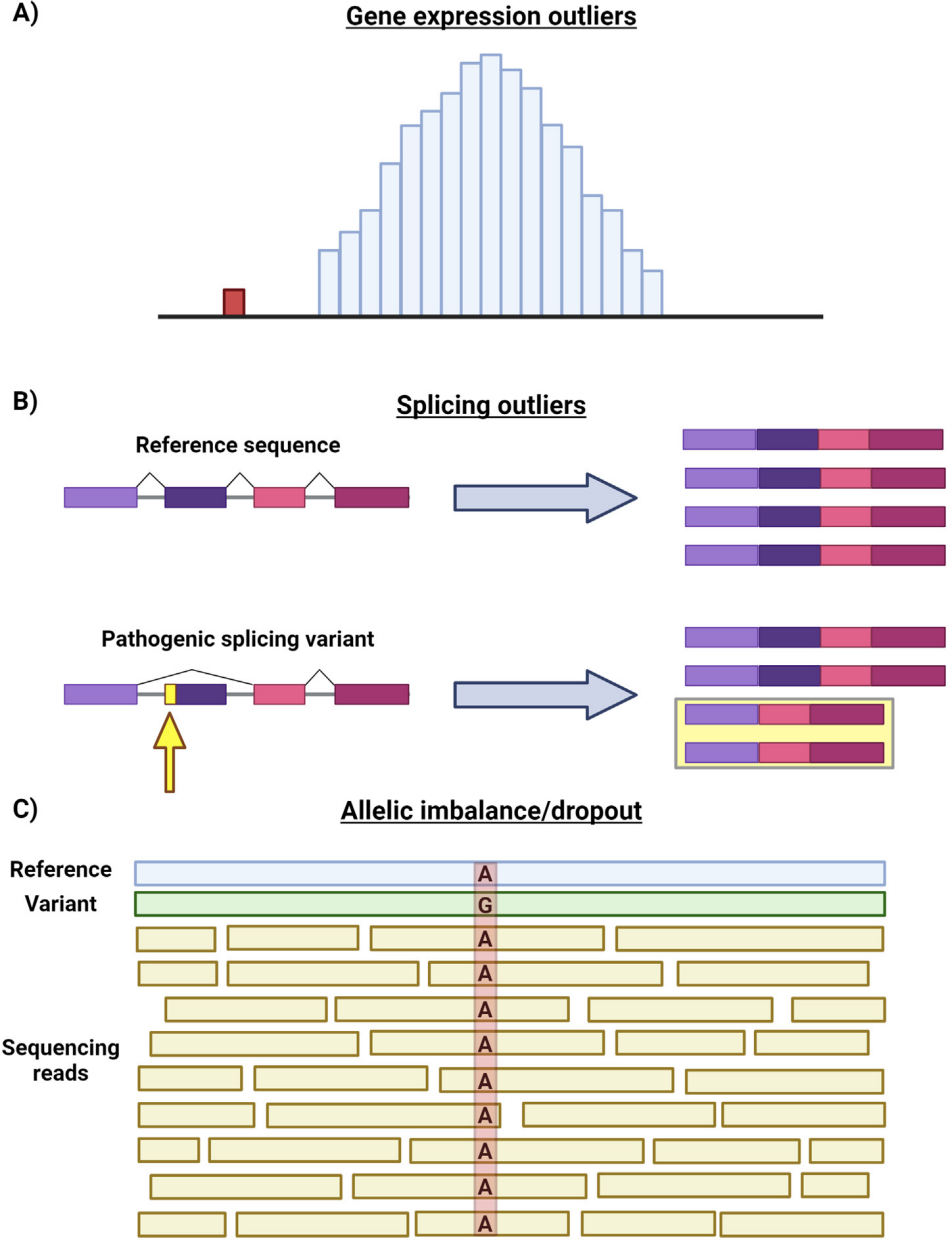
**Transcriptomic approaches to mitochondrial neurodevelopmental disorders.** RNA sequencing (RNA-seq) can identify gene expression outliers, splicing outliers, and allelic imbalance/dropout within cells, tissues, or other biospecimens. A) Normal gene expression within cells or tissues is established by performing RNA-seq on control samples (*blue bars*). Each bar represents gene expression levels within a sample. Gene expression within the sample (*red bar*) is found to be an outlier. The lower expression within the sample indicates that a genetic variant reducing gene expression may be present within the gene or gene regulatory elements. B) RNA splicing results in the removal of intronic sequences and the splicing of exons (*colored rectangles*). Abnormal splicing patterns (splicing outliers) may result from pathogenic splicing variants; these can be detected in comparison to control samples. The pathogenic splicing variant in the figure results in exon skipping in half of transcripts (*yellow box*). C) The relative expression of two alleles at a particular locus can be quantified by examining how many reads contain a single nucleotide variant or polymorphism versus the reference sequence. Pathogenic variation within a gene or gene regulatory element that reduce one allele's expression will result in allelic imbalance or dropout as shown in the figure. Figure created using Biorender.com.

RNA sequencing (RNA-seq) provides a relatively unbiased survey of all transcripts through which aberrant gene expression, aberrant splicing, and allelic dropout/imbalance can be detected (Fig. 3) [12,[65], [66], [67]]. Gene expression outliers within samples can be identified by comparison with control samples (Fig. 3A); increased or decreased gene expression may point to a genetic variant disrupting a promoter or enhancer. Genetic variants which introduce premature termination codons (PTC) may reduce gene expression via transcript degradation due to the quality control mechanism nonsense-mediated decay (NMD). Similarly, splicing outliers including exon skipping and intron inclusion can be detected (Fig. 3B); these outliers may occur due to genetic variants which destroy or introduce novel splice sites or alter exonic and intronic splicing enhancers and silencers. Finally, RNA-seq can detect the preferential expression of a single allele, so-called allelic imbalance or dropout (Fig. 3C). Allelic imbalance may result from genetic variants in regulatory regions or genetic variants which introduce PTCs and trigger NMD.

RNA-seq may be performed in bulk using tissue specimens like muscle biopsies or cell cultures or at the single cell level. Single-cell transcriptomics has provided an unprecedented window into the heterogeneity and complexity within tissues and cells and is a very active area of investigation [68]. Single-cell transcriptomics has been used to study the signaling pathways and cell types involved in mitochondrial disease pathogenesis [69,70].

Bulk RNA-seq using patient blood samples, fibroblasts and muscle tissue has been shown to boost diagnostic rates in rare Mendelian disorders. Characterization of gene expression outliers, allelic dropout/imbalance, and aberrant splicing via RNA-seq can help identify pathogenic genetic variants missed during the analysis of ES and GS data (Fig. 3) [42,[65], [66], [67],[71], [72], [73]]. RNA-seq can also functionally validate intronic variant pathogenicity through demonstration of splicing abnormalities. For example, transcriptomics identified the mitochondrial disease gene *TIMMDC1* through recognition of a homozygous deep intronic variant introducing a pseudoexon and premature termination codon [72]. Moreover, RNA-seq can identify the genes and pathways impacted by gene knockout/knockdown or pathogenic genetic variation and thereby provide insights into the biology underlying mitochondrial neurodevelopmental disorders. Thus, transcriptomics is an important component of functional genomics toolkit.

Transcriptomic data interpretation remains challenging for several reasons. First, each tissue has distinctive patterns of gene splicing and expression. Comparisons between tissues are therefore inherently limited. Important genes and transcripts in the brain may not be present in clinically accessible tissues like blood, fibroblasts, or skeletal muscle. Second, sex, age, genotype, and other health conditions influence transcriptomics, and age- and sex-matched healthy control samples and data are seldom available. Third, standardized approaches to transcriptomic data analysis remain in development. Finally, nearly all transcriptomic studies to date have utilized short-read RNA-seq. The short sequencing reads of SRS cannot capture the entire coding sequence of most genes; therefore, transcript analysis requires computationally reconstruction of each transcript and is believed to lack many isoforms [74]. LRS can capture the entire coding sequence of transcripts and may therefore provide a more accurate transcriptomic survey, but its high-cost limits coverage and may limit sensitivity for detection of low frequency but biologically meaningful isoforms [74]. Alternative approaches to study splicing dysfunction like high throughput splicing assays are in development [64].

### Proteomics

**Fig. 4:**
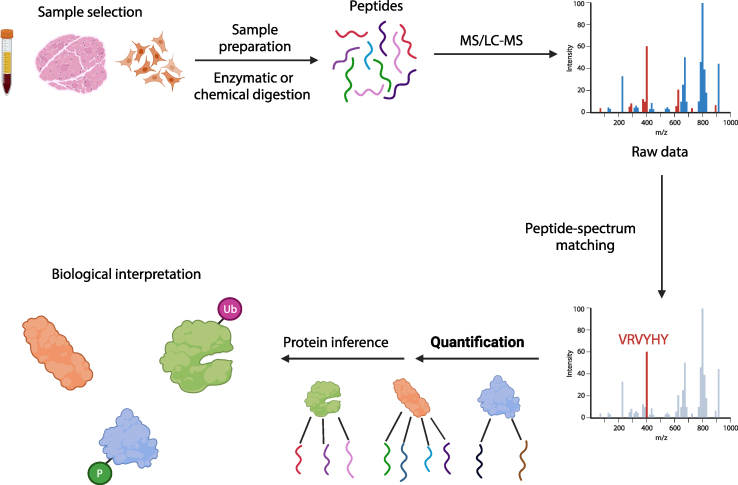
**Proteomic approaches to mitochondrial neurodevelopmental disorders**. Summary of a bottom-up proteomic study. Proteomics can be performed on biospecimens, tissue samples, or cell lines (*sample selection*). Samples are then enzymatically or chemically digested to generate short peptides. These peptides then undergo MS, LC-MS, or HPLC-MS. Raw data then undergoes peptide-spectrum matching to identify the sequence of each peptide. Individual peptides are then quantified, and peptide fragment sequences are compared with the sequences of known proteins; the quantified proteins are then biologically interpreted. Figure created using Biorender.com.

Biological complexity increases at each step along the central dogma: while the human genome contains approximately 20,000 genes, the human transcriptome and proteomes are estimated to contain approximately 300,000 transcripts and 10^6^–10^9^ protein isoforms, respectively [75]. The large jump in complexity from transcriptome to proteome reflects post-translational modifications, *e.g.*, glycosylation, phosphorylation, ubiquitination, and protein cleavage. Comprehensive characterization of the proteome can be achieved through mass spectroscopy (MS) (Fig. 4) [76,77]. MS permits the quantification of posttranslational modifications and protein isoforms within biospecimens, cells, and tissues. The combination of liquid chromatography (LC) or high-performance liquid chromatography (HPLC) to separate complex mixtures of proteins with MS is particularly suitable for high-throughput proteomics.

There are three main MS proteomic strategies: top-down, bottom-up, and shotgun approaches [77]. The top-down approach involves fragmentation of full-length proteins within the mass spectrometer followed by quantification of fragment sizes. In the bottom-up approach, proteins are enzymatically or chemically digested before performing MS. Finally, in the shotgun approach, complex solutions like serum, urine, and cell or tissue lysates are fragmented followed by HPLC before MS.

MS has also been applied to single-cell proteomics [78]. Like single-cell transcriptomics, single-cell proteomics provides an opportunity to dissect cellular heterogeneity in mitochondrial neurodevelopmental disorders. However, single-cell proteomics faces significant technical challenges [78]. While transcripts can be amplified by polymerase chain reaction (PCR) to facilitate single-cell transcriptomics, an analogous process for proteins does not exist. Thus, sample preparation must be carefully optimized to limit protein loss. The throughput of current single-cell proteomic approaches is also limited but is enhanced by multiplexed analysis. Other proteomic approaches including protein pathway array, proximity extension assay, and single-molecule protein sequencing exist but are beyond the scope of this review [77,79].

High-throughput proteomics thus has the potential to identify biomarkers useful for mitochondrial neurodevelopmental disorders diagnosis and assessment of response to therapeutic interventions. It also has the potential to identify biomarkers to inform the development of novel therapeutic modalities. Proteomics can also aid in the clinical diagnostic interpretation of single nucleotide variants, indels, and structural variants in mitochondrial neurodevelopmental disorders by providing a functional readout of protein stability and post-translational modification state [12]. An important limitation of MS is that most missense variants do not alter protein stability and abundance. Unfortunately, the integration of unbiased, high-throughput proteomics into mitochondrial neurodevelopmental disorders or any rare Mendelian disorders has been limited to date.

### Metabolomics

Metabolomics is the systematic characterization of small molecule metabolites within tissues, cells, or biospecimens like plasma, urine, or cerebrospinal fluid [80,81]. Multiple analytical platforms exist for metabolomic studies including MS and nuclear magnetic resonance (NMR) [82]. MS is widely used in metabolomics: it has greater sensitivity than NMR, is less expensive, and has a broader detection range. However, it requires sample manipulation prior to analysis and results in sample destruction. MS is typically coupled with liquid or gas chromatography to reduce complexity and improve sample throughput and sensitivity. Advantages of NMR include high reproducibility, rapid analytic time, and no need for sample manipulation prior to analysis. Additionally, NMR preserves samples for downstream analysis. The major downside of NMR compared with MS is lower sensitivity. The detection of low abundance compounds with NMR is particularly challenging. At present, neither technology can characterize all possible metabolites; the choice of technology must be tailored to each application [82].

There are two approaches to metabolomics: targeted and untargeted metabolomics [83,84]. Targeted metabolomics strategies involve the quantification of a defined set of metabolites, whereas untargeted metabolomics quantify all metabolites within a sample. Targeted metabolomics encompasses traditional clinical diagnostic metabolic studies like plasma amino acids and urine organic acids. Targeted approaches generally result in the absolute quantification of metabolite levels and have reduced false positives and artifacts than untargeted approaches. Thus, targeted metabolomics is ideal for testing hypotheses about specific metabolites or metabolic pathways. Targeted metabolomics is not ideal if the metabolic pathway of interest is not fully understood. Furthermore, targeted metabolomics is typically limited to a relatively small number of metabolites. Untargeted metabolomics can provide data about 100s–1000s of small molecule metabolites within a sample and therefore can be very helpful for non-hypothesis driven investigational research. Disadvantages of untargeted metabolomics include semi-quantitative measurements of metabolites and greater susceptibility to false positives and artifacts than targeted metabolomics.

Untargeted metabolomic approaches using liquid chromatography-mass spectroscopy (LC-MS) have recently entered the clinical realm [80]. In patients with inborn errors of metabolism (IEM), untargeted metabolomics has superior diagnostic ability compared to traditional metabolic testing [85]. Untargeted metabolomics has been applied to multiple biospecimens including plasma, urine, and cerebrospinal fluid. As many metabolites are unstable or labile, careful sample preparation and storage is critical. A systematic analysis of metabolomics utility in mitochondrial neurodevelopmental disorders has not been performed, but the broader range of metabolites surveyed may provide superior sensitivity over traditional metabolic testing. A distinctive metabolomic signature for NAXE and NAXD deficiency including abnormalities in nicotinamide metabolites, branched-chain amino acids, and lipids was reported [86,87]. Resolution of these abnormalities in NAXE and NAXD deficiency with niacin supplementation suggests metabolomics may also be a useful therapeutic biomarker in these conditions and perhaps other mitochondrial neurodevelopmental disorders.

### Multiplexed assays of variant effect and saturation genome editing

**Fig. 5:**
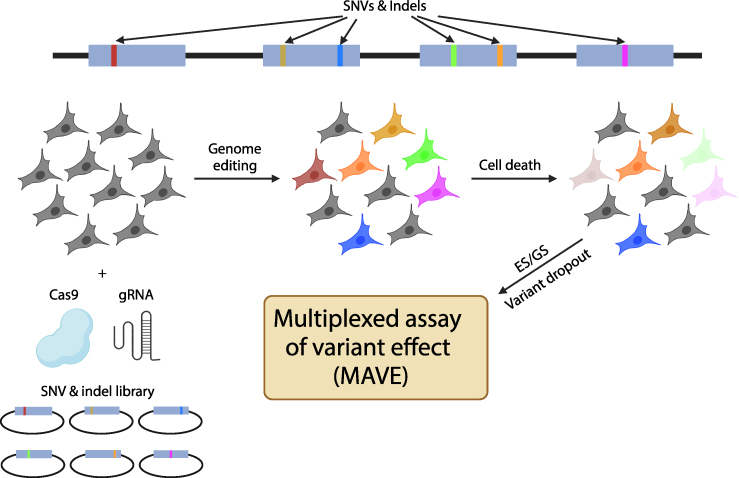
**Saturation Genome Editing and Multiplexed Assays of Variant Effect.** Saturation Genome Editing (SGE) involves the introduction of all possible single nucleotide variants (SNVs) and indels into a gene or locus of interest (*top half*) into cell lines using CRISPR-Cas9. The experimental process is described in the bottom half of the figure. The cell line HAP1 is often used for genome editing since it contains a haploid genome. An SNV or indel library and CRISPR-Cas9 is introduced into cells to induce homology directed repair. Each cell acquires a single mutation. If the gene is essential for cellular survival, genetic variants resulting in a null allele will cause cell death. The cell population is subsequent sequenced by ES or GS. The dropout of variants within the population indicates that the variant is pathogenic. The degree of variant depletion is calculated and used to generate a functional score. Multiplexed assays of variant effect (MAVE) thus consist of the functional score of each variant assayed in an SGE study.

Traditional approaches to study genetic variation's impact on mitochondrial function utilize patient samples or genetic manipulation of cell lines including transgene expression or gene editing. While these approaches offer important insights into mitochondrial gene function, they are not readily translated to high throughput screens capable of resolving the consequences of all possible genetic variation within a single gene, locus, or gene family. The development and optimization of highly efficient genome editing techniques like CRISPR-Cas9 has finally made this a reality through multiplexed assays of variant effect (MAVE) and saturation genome editing (SGE) (Fig. 5) [[88], [89], [90]]. SGE utilizes CRISPR/Cas9-mediated homology directed repair to introduce genetic variants into a gene or locus at scale. To date, SGE has primarily been performed using the haploid cell line HAP1. The introduction of gene-disrupting genetic variants into essential genes leads to their depletion in culture over time, whereas benign genetic variants persist. Each variant can thus be scored based on its persistence in culture. The approach is most sensitive for null alleles (*e.g.*, loss-of-function variants), although hypomorphic alleles may cause slower but appreciable variant depletion [90]. The functional scores for each variant form an atlas which helps resolve variants of uncertain significance (VUS) into benign versus pathogenic. SGE also provides a map of essential domains, amino acids, and nucleotides within genes. Although most SGE studies have focused on cellular viability, other approaches using high-throughput imaging readouts of mitochondrial content or morphology can be conceived [[91], [92], [93]]. While MAVE studies of mitochondrial genes have not been published, several are ongoing (https://www.mavedb.org/). As many nuclear mitochondrial genes are essential, they should be good targets for SGE [94].

Unfortunately, the implementation of SGE for mitochondrial genome is technically challenging. Challenges in mitochondrial genome editing include 1) multiple copies of the mitochondrial genome per cell, 2) lack of homology directed repair in mitochondria, 3) the mitochondrial membrane as a barrier, and 4) RNA import into mitochondria [95]. Recent reports of successful base editing of the mitochondrial genome, an approach which does not require homology directed repair, provide an important step toward mitochondrial genome SGE [96].

### Complete functional annotation of the human genome requires global data aggregation

**Fig. 6:**
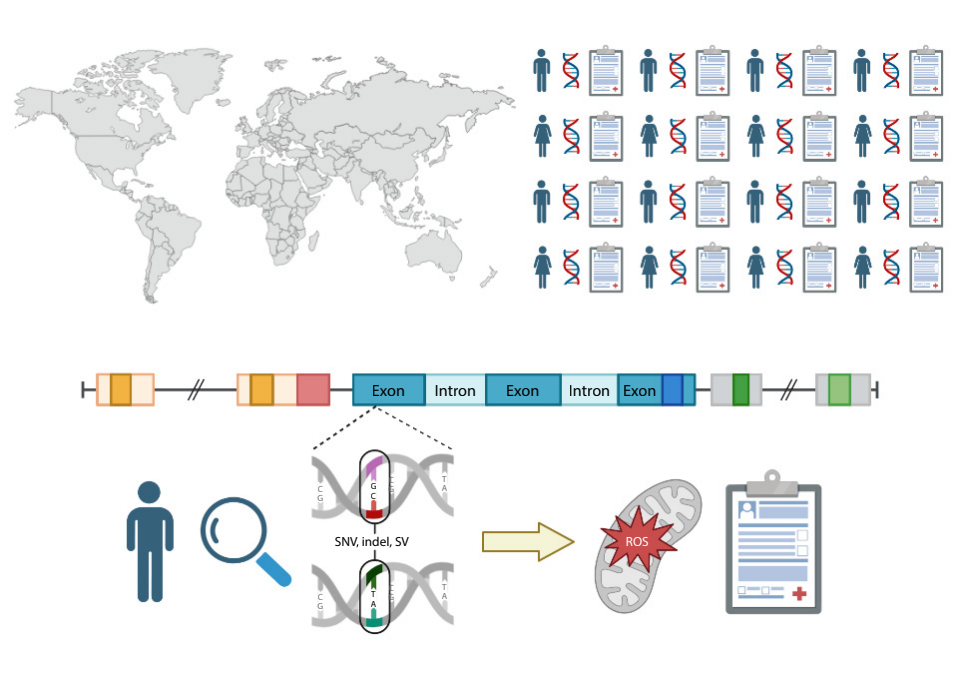
**Functional annotation of the human genome through global data aggregation.** The integration and aggregation of individual-level genomic and phenotypic data derived from health records (*top half*) on a global scale is necessary to provide full functional annotation of the human genome. Such efforts are necessary to fully realize the promise of precision medicine through accurate prediction of mitochondrial dysfunction and mitochondrial neurodevelopmental disorders from an individual's genotype (*bottom half*). Figure created using Biorender.com.

While the completion of the Human Genome Project in April 2003 was a major milestone in human genetics and medicine, it was only the beginning of vast, ongoing global effort to catalog and functionally annotate all possible human genetic variations. Due to rapid population growth over the last century, it is estimated nearly all possible single nucleotide variants exist within the human population [97]. Thus, humanity has engaged in a massive saturation mutagenesis experiment [97]. While models of human genetic variation in non-human organisms and cell lines have been helpful, the translation of human genetics into these systems is imperfect at best. The ultimate proof of a genetic variant's phenotypic impact is the recurrent association of a variant with a phenotype in humans (Fig. 6).

Allelic series – the collection of variant alleles at a gene or genomic locus which generate a range of phenotypic traits – are an essential component of functional genome annotation [98]. Examples of mitochondrial genes with evolving and complex allelic series include *HPDL*, *NAXD,* and *NAXE*. *HPDL* encodes the mitochondrial protein 4-hydroxyphenylpyruvate dioxygenase-like [[99], [100], [101]]. Although it shares significant sequence similarly with the tyrosine catabolic enzyme 4-hydroxyphenylpyruvate dioxygenase, it is not involved in tyrosine catabolism but instead functions within the CoQ10 biosynthesis pathway [102]. Biallelic variants in *HPDL* cause a mitochondrial neurodevelopmental disease spectrum ranging from neonatal mitochondrial encephalopathy and Leigh syndrome to adolescent-onset, isolated hereditary spastic paraplegia (HSP) [[99], [100], [101]]. While genotype-phenotype correlations were not initially apparent, subsequent expansion of the allelic series demonstrates *HPDL* loss-of-function variants associate with more severe phenotypes in keeping with a recessive disease model [101]. Furthermore, a likely Latino founder allele, *HPDL*: c.859T>C p.Y287H, shows a strong genotype-phenotype correlation with simple HSP in the homozygous state (*D. Calame, personal observation*).

*NAXD* and *NAXE* encode NAD(P)HX dehydratase and NAD(P)HX epimerase, respectively [103,104]. The two enzymes form a two-step pathway for the detoxification of hydrated, toxic forms of NAD(P)H. As hydrated NAD(P)H accumulates during cellular stress, deficiency of either enzyme results in metabolic crises triggered by fever: progressive, early-onset encephalopathy with brain edema and/or leukoencephalopathy (PEBEL) [[103], [104], [105]]. While initial publications described severe metabolic decompensation and death in infants or toddlers, the evolving *NAXE* and *NAXD* allelic series demonstrates a wider phenotypic spectrum with some individuals surviving crises, lacking cardinal clinical features like skin rashes, or even having adolescent-onset [86,87]. While genotype-phenotype correlations remain under study, there is preliminary evidence that pathogenic variants early in *NAXD* may associate with milder phenotypes [86]. A potential explanation for this genotype-phenotype correlation is *NAXD* encodes distinct cytosolic and mitochondrial isoforms; early mutations should only impact the cytosolic isoform and therefore preserve mitochondrial NAXD activity [86].

Functional human genome annotation is also uncovering genetic mimics of mitochondrial neurodevelopmental disorders. An illustrative example is *SNAPC4* [106]. *SNAPC4* encodes a component of the small nuclear RNA (snRNA)-activating protein complex. The snRNA-activating complex directs snRNA transcription and is consequently required for spliceosome assembly and proper RNA splicing. Biallelic pathogenic variation in *SNAPC4* decreases snRNA expression and leads to global splicing dysregulation. While most individuals with *SNAPC4*-related disorders have non-specific neurodevelopmental disorders characterized by developmental delay and movement disorders, some have severe infantile-onset neurologic dysfunction with bilateral basal ganglia T2 hyperintensity and a clinical diagnosis of Leigh syndrome. Other causes of bilateral infantile striatal necrosis without evidence of mitochondrial dysfunction include *NUP62*, *VAC14*, and *ADAR1* [[107], [108], [109]]. Identification of these mimics is critical. Phenotypic and genetic heterogeneity are likely a barrier to success in mitochondrial disease clinical trials; the exclusion of non-mitochondrial genetic mimics is needed to maximize the likelihood of trial success. Furthermore, treatments optimizing mitochondrial function or targeting mitochondrial dysfunction are unlikely to benefit patients with non-mitochondrial disorders.

Realizing the full promise of the human genomics at scale remains a massive undertaking. The functional annotation of common genetic variation through genome-wide association studies (GWAS) is nearly complete. As common variant studies have concentrated in Europe and the United States due to global inequities, remaining common variant studies must concentrate on the Global South and other understudied populations [110]. In contrast, the study of rare genetic variation has only just begun. An explosion of neurodevelopmental disease-gene associations has resulted from family-based studies, next generation sequencing, and online gene matchmaking [4,[111], [112], [113], [114], [115], [116], [117], [118], [119], [120], [121], [122], [123], [124], [125], [126], [127], [128]], but genome-wide functional annotation requires greater international integration of human genomic and phenotypic data. National programs like *UK Biobank* and *All of Us* represent an important step in this direction [129,130], but a wealth of genomic and phenotypic data remains locked away in data silos: research databases, clinical diagnostic laboratories, and electronic health records. Ultimately, the complete functional annotation of the human genome requires greater global data sharing with societal commitment for the benefit of all.

### Challenges inherent to multi-omic approaches

While considerable progress has been made towards the identification of the genes and genetic variants responsible for mitochondrial neurodevelopmental disorders, a definitive genetic diagnosis is obtained through ES or GS in less than half of patients [12,131]. There are several potential explanations for inconclusive ES/GS [41]. First, many disease-gene associations clearly remain to be discovered. The pace of gene discovery is rapid, yet as of December 8th, 2023, only 4868 of the approximately 20,000 computationally-annotated protein coding genes have phenotypic associations in the Online Mendelian Inheritance in Man (OMIM) database (https://omim.org/). Second, perceived phenotypic mismatch and phenotypic expansions can result in a failure to obtain a definitive diagnosis. Phenotypic data guides the analysis of genomic data as the genetic diagnosis is expected to match the clinical synopsis of the associated disease trait. In some cases, clinical phenotyping data is extracted into structured ontology terms (*e.g.*, Human Phenotype Ontology) which then feeds into variant filtering algorithms [[132], [133], [134]]. Therefore, incorrect or limited clinical phenotypic data may result in the filtering out of a pathogenic variant and therefore a “negative” result. Misinterpretation of phenotypic data can also play a role. For example, hypotonia is often misconstrued as a sign of neuromuscular (*i.e.*, muscle or peripheral nerve) disease but is just as likely to have a central nervous system origin in mitochondrial neurodevelopmental disorders. Phenotypic expansion refers to the extension of the phenotypic spectrum associated with a particular gene or locus [135]. It is now recognized most genes associate with a phenotypic spectrum ranging from mild to severe disease due to gene dosage effects in haploinsufficient or recessive disease models (*e.g.*, hypomorphic missense variant allele versus null alleles) or allele-specific disease mechanisms (*e.g.*, loss-of-function variants and haploinsufficiency versus missense variants and gain of function). However, the phenotypic spectrum can be so broad as to limit recognition of allelic disorders, *e.g.*, biallelic *HPDL* variants associating with Leigh syndrome on the severe end of the spectrum and simple hereditary spastic paraplegia on the mild end. Third, technical limitations may prevent a definitive diagnosis. For example, ES may struggle to detect small copy number variants (*e.g.*, single or multi-exon deletions and duplications) and will generally not detect pathogenic noncoding variants. Forth, there may be inadequate evidence to interpret the pathogenicity of a variant. This can reflect challenges in variant interpretation due to limitations of *in silico* predictive programs but also may be due to absence of family genomic data to determine *de novo* status or to test whether variants segregate according to Mendelian expectations. This is especially true for noncoding variation as *in silico* tools capable of evaluating their functional impact remain in a nascent stage. Furthermore, as population genomic databases which do not contain individual-level phenotypic data increase in size by ingesting data from large-scale programs like UK Biobank, All of US, 100,000 Genomes Project, and Regeneron Genomics Center which do not screen out individuals with severe neurodevelopmental disorders, there may be new challenges in the interpretation of ultra-rare genetic variants in a Mendelian context [129,130,136,137].

There are many limitations to multi-omics including cost, a requirement for highly specialized knowledge, and logistical challenges. Collaboration and team science are a requirement for large-scale multi-omic studies. Fortunately, costs continue to drop with technical advances. The cost of GS has dramatically declined from the time of the Human Genome Project (∼$3 billion US dollars) to the present era of $100 genomes [27,138]. Another important limitation is a lack of platforms for the integrated analysis of multi-omic data. The open source *seqr* software platform allows for integrative analysis of genomic and transcriptomic data; efforts to incorporate proteomic data are ongoing [139]. Further advances are needed to fully realize the potential of functional genomics in mitochondrial neurodevelopmental disorders.

## Small molecule therapeutics in mitochondrial neurodevelopmental disorders

We live in an exciting era. RNA/DNA therapies like antisense oligonucleotides (ASOs), small interfering RNAs (siRNAs), gene replacement therapies, and genome editing are entering the clinical realm with proven therapeutic benefit and life extension for rare, previously fatal Mendelian diseases [140,141]. Yet with ∼20,000 protein-coding genes and the considerable genetic heterogeneity of mitochondrial neurodevelopmental disorders, RNA/DNA therapeutics will remain inaccessible to most patients. However, it is important to recognize other therapeutic strategies like small molecules can be highly efficacious (Table 1). Indeed, small molecule therapeutics should be considered for all patients with mitochondrial neurodevelopmental disorders, even those without established small molecule treatments, by conceptualizing the gene defect within the context of the underlying biological pathway. Here we provide several examples of small molecule therapeutics for mitochondrial neurodevelopmental disorders.

**Table 1:** Small molecule therapeutics for mitochondrial neurodevelopmental disorders.

Condition	OMIM #	Gene(s)	Treatment
Progressive early-onset encephalopathy with brain edema and/or leukoencephalopathy 1	617186	*NAXE*	Niacin
Progressive early-onset encephalopathy with brain edema and/or leukoencephalopathy 2	618321	*NAXD*	Niacin
Coenzyme Q10 deficiency	607426, 616276, 614650, 616733, 614654, 612016, 615573, 614651, 614652	*COQ2, COQ4, COQ6, COQ7, COQ9, ADCK3, ADCK4, PDSS1, PDSS2*	CoQ10
Biotin-thiamine-responsive basal ganglia disease	607483	*SLC19A3*	Thiamine, biotin
Thiamine pyrophosphokinase deficiency	614458	*TPK1*	Thiamine, biotin
Thiamine-responsive pyruvate dehydrogenase deficiency	312170	*PDHA1*	Thiamine
Multiple acyl-CoA dehydrogenase deficiency	231680, 616839, 255100	*ETFA, ETFB, ETFDH, SLC25A32, FLAD1*	Riboflavin
ACAD9 deficiency	611126	*ACAD9*	Riboflavin
Molybdenum cofactor deficiency	252150, 252160, 615501	*MOCS1, MOCS2, GPHN*	Fosdenopterin
Biotidinase deficiency	253260	*BTD*	Biotin
Holocarboxylase synthetase deficiency	253270	*HLCS*	Biotin
*HPDL*-related disorders	619026, 619027	*HPDL*	?CoQ10, ?4-HMA
Mitochondrial interferonopathies	614932, 614934, 608703, 617183, 618810	*PNPT1*, *ATAD3A*, *SUPV3L1*	?Jak kinase inhibition

OMIM, Online Mendelian Inheritance in Man (www.omim.org); ? Theoretical small molecule therapeutics with limited supporting clinical data.

### Coenzyme Q10 deficiencies and HPDL-related disorders

Coenzyme Q10 (CoQ10) is an integral cofactor in mitochondrial oxidative phosphorylation. Several genes are involved in CoQ10 biosynthesis (Table 1). Primary CoQ10 deficiency typically manifests with brain, muscle, and kidney involvement like encephalomyopathy, cerebellar ataxia, severe multisystem infantile disease, or steroid-resistant nephrotic syndrome [142]. CoQ10 supplementation results in clinical stabilization and even symptom reversal in some but not all individuals with primary CoQ10 deficiency [142]. Intriguingly, hydroxyphenylpyruvate dioxygenase-like (HPDL) was recently found to produce the CoQ10 intermediate 4-hydroxymandelate (4-HMA) [102]. As biallelic pathogenic variation in *HPDL* causes mitochondrial neurodevelopmental disorders ranging from Leigh syndrome to simple hereditary spastic paraplegia, supplementation of CoQ10 or 4-HMA might have therapeutic benefit in these conditions [[99], [100], [101]]. A natural history study of *HPDL*-related disorders (ClinicalTrials.gov ID: NCT05848271) is ongoing with the end goal of a clinical trial of 4-HMA supplementation.

### Thiamine, biotin, and SLC19A3/TPK1 deficiency

*SLC19A3* is a member of the solute carrier (SLC) family and encodes a thiamine transporter [143]. Individuals with biallelic pathogenic *SLC19A3* variants develop infantile or early childhood encephalopathy and Leigh syndrome [143]. *TPK1* encodes thiamine pyrophosphokinase, an enzyme which converts thiamine into the active cofactor thiamine pyrophosphate [144]. Thiamine pyrophosphate deficiency similarly causes episodic encephalopathy and Leigh-like syndromes [144]. While some patients with SLC19A3 or TPK1 deficiency respond to biotin supplementation, most evidence supports thiamine supplementation as the definitive treatment [144,145]. Early intervention and long-term supplementation is critical. Neurologic injury following encephalopathic crisis may be irreversible, and recurrence after discontinuation of thiamine therapy has been reported [146]. Substrate supplementation should be considered for other disorders of SLC transporters; increased substrate concentrations may boost substrate uptake through hypomorphic transporters or transport through alternative, low-affinity transporters.

### Niacin and NAXE/NAXD deficiency

NAXE and NAXD provide a two-step mechanism for the detoxification of hydrated NAD(P)H (NAD(P)HX) [103,104]. Children with NAXE or NAXD deficiency develop encephalopathy and brain edema triggered by febrile illness or other metabolic stressors. Lactate in CSF or plasma may be elevated or normal. Pellagra-like skin lesions have observed in NAXE-related crises. The morbidity and mortality of crises is high. As NAXE/NAXD deficiency is expected to cause NAD(P)HX accumulation and NAD+ pool depletion, trials of prophylactic niacin supplementation to replete NAD+ pools has been performed in several individuals with prolonged survival without crisis recurrence and improvement in neurologic symptoms [86,87]. Metabolomic testing during NAXE/NAXD crises supports the NAD+ pool depletion hypothesis, and repeat testing post-niacin supplementation showed metabolic normalization [86,87]. NAD+ deficiency has also been identified in adults with mitochondrial myopathies, and niacin repletion increased blood and muscle NAD+ levels and improved muscle strength and mitochondrial biogenesis [147]. Systematic study of NAD+ levels in individuals with mitochondrial neurodevelopmental disorders is needed to determine whether niacin supplementation might be beneficial more broadly in this population.

### Jak inhibition and type 1 interferonopathies

*ATAD3A* encodes the ATPase family AAA-domain containing protein 3A, a nuclear-encoded mitochondrial membrane protein involved in mtDNA organization and mitochondrial dynamics [148]. Monoallelic and biallelic pathogenic variants in *ATAD3A* cause mitochondrial neurodevelopmental disorders: autosomal dominant and recessive Harel-Yoon syndrome and autosomal recessive pontocerebellar hypoplasia [[148], [149], [150], [151]]. Recent expansion of the *ATAD3A* allelic series demonstrates some individuals with Harel-Yoon syndrome due to dominant negative *ATAD3A* variants exhibit autoinflammation/autoimmunity and elevated type 1 interferon activity [152]. Loss of ATAD3A in Harel-Yoon syndrome results in autoinflammation due to cytosolic mtDNA leakage. Cytosolic mtDNA is recognized as ‘foreign’ by DNA sensors cGAS/STING which activate the type 1 interferon pathway. A similar phenomenon has been identified in individuals with *PNPT1*-related disorders [153]. Furthermore, pathogenic variation in *SUPV3L1*, a gene encoding an RNA helicase involved in the mitochondrial degradosome, results in double-stranded RNA (dsRNA) accumulation, type 1 interferon activation, mitochondrial dysfunction, and white matter disease [154,155]. Like cytosolic mtDNA, dsRNA is recognized by cellular sensors including MDA5, RIG-1, TLR7/8. TLR3, PKR, and OAS1-3 which also trigger the type 1 interferon pathway. Excess type 1 interferon is the hallmark of Aicardi-Gouiterres syndrome (AGS), a genetic neurodevelopmental autoinflammatory disorder resulting from dysregulated type 1 interferon expression and signaling [156]. A direct overlap between AGS and severe mitochondrial neurodevelopmental disorders was suggested in a case report of a patient with AGS and a homozygous *PNPT1* missense variant [157]. As excess type 1 interferon drives neurodegeneration in AGS, it is possible but yet unproven that type 1 interferon may also contribute to the pathogenesis of mitochondrial neurodevelopmental disorders. This could potentially have therapeutic implications as Janus kinase inhibitors suppress type 1 interferon signaling and have demonstrated therapeutic benefit in AGS. Jak inhibition may also be beneficial for autoinflammation/autoimmunity in mitochondrial neurodevelopmental disorders.

## Conclusions and perspectives

Progress in mitochondrial disease mechanisms and therapeutics has been hampered by genetic heterogeneity, an ever-widening clinical spectrum, and non-mitochondrial disease genetic mimics. The diagnostic rate in suspected mitochondrial neurodevelopmental disorders remains low [158]. However, functional genomic approaches – short and long-read genomic sequencing, transcriptomics, proteomics, metabolomics, saturation genome editing, and global genotype-phenotype data aggregation – represent a critical step towards the comprehensive functional annotation of all genes and genomic regions involved in mitochondrial disease. These advances suggest the bold prediction of the United States National Human Genome Research Institute (NHGRI) that “the biological function(s) of every human gene will be known” and “the clinical relevance of all encountered genomics variants will be readily predictable, rendering the diagnostic designation ‘variant of uncertain significance (VUS)’ obsolete” by 2030 may be achievable [159]. Furthermore, the insights into mitochondrial disease pathogenesis resulting from these studies will provide a platform for development of better disease biomarkers, small molecule medicines, and RNA/DNA therapies.

While clinical genomics has brought precision diagnosis to many individuals with mitochondrial neurodevelopmental disorders, the implementation of precision medicine has been slow. Precision medicine refers to the development of individualized medical plan based on genotype, phenotype, and environment. A major barrier to precision medicine in mitochondrial neurodevelopmental disorders is their rarity. With many conditions identified only in a single family or a few families, it is not yet possible to identify genotype-phenotype correlations which may inform prognostication. Their rarity also provides a limit to the natural history data available to inform prognostication. Ascertainment bias also plays a role as comprehensive genetic investigations like ES only became available in the clinic in 2011 and are primarily performed on children in most healthcare systems; therefore, there is a paucity of clinical records available for adults to inform our understanding of the long-term trajectory and sequelae of most mitochondrial neurodevelopmental disorders.

One way to address precision medicine in mitochondrial neurodevelopmental disorders is through the implementation of artificial intelligence (AI) and machine learning (ML). The integration of large datasets like electronic health records, genomic databases, and high-throughput multi-omic investigations provides an ideal opportunity for the application of AI/ML to mitochondrial neurodevelopmental disorders. AI/ML approaches have already been applied to metabolomic data within UK Biobank to identify correlations between metabolomic states and common diseases [160]. AI programs have also been shown to accurately predict risk of cardiac disease and cancer [161]. The rich datasets generated for individuals with mitochondrial neurodevelopmental disorders – neuroimaging, clinical phenotyping, genotyping, and potentially multi-omic technologies – may provide an opportunity to use AI to improve precision medicine. Finally, AI may provide opportunities to quickly identify biological pathways and FDA-approved drugs for drug repurposing in mitochondrial neurodevelopmental disorders [162]. A future in which precision medicine is a reality for all patients with mitochondrial neurodevelopmental disorders is on the horizon.

## Author contribution

Daniel Grant Calame (DGC) wrote the manuscript and designed the figures. Lisa T. Emrick (LTE) helped write the manuscript and provided critical feedback.

## Declaration of competing interest

The authors declare that they have no known competing financial interests or personal relationships that could have appeared to influence the work reported in this paper.

## References

[1] Straub L., Bateman B.T., Hernandez-Diaz S., Diaz S., York C., Lester B (2022). Neurodevelopmental disorders among publicly or privately insured children in the United States. JAMA Psychiatr.

[2] Faraone S.V., Larsson H. (2019). Genetics of attention deficit hyperactivity disorder. Mol Psychiatr.

[3] Parenti I., Rabaneda L.G., Schoen H., Novarino G. (2020). Neurodevelopmental disorders: from genetics to functional pathways. Trends Neurosci.

[4] Mitani T., Isikay S., Gezdirici A., Gulec E.Y., Punetha J., Fatih J.M. (2021). High prevalence of multilocus pathogenic variation in neurodevelopmental disorders in the Turkish population. Am J Hum Genet.

[5] Jansen S., Vissers L.E.L.M., de Vries B.B.A. (2023). The genetics of intellectual disability. Brain Sci.

[6] Khacho M., Harris R., Slack R.S. (2019). Mitochondria as central regulators of neural stem cell fate and cognitive function. Nat Rev Neurosci.

[7] Rangaraju V., Lewis T.L., Hirabayashi Y., Bergami M., Motori E., Cartoni R. (2019). Pleiotropic mitochondria: the influence of mitochondria on neuronal development and disease. J Neurosci.

[8] Herculano-Houzel S. (2011). Scaling of brain metabolism with a fixed energy budget per neuron: implications for neuronal activity, plasticity and evolution. PLoS One.

[9] Nasca A., Scotton C., Zaharieva I., Neri M., Selvatici R., Magnusson O.T. (2017). Recessive mutations in MSTO1 cause mitochondrial dynamics impairment, leading to myopathy and ataxia. Hum Mutat.

[10] Al Ojaimi M., Salah A., El-Hattab A.W. (2022). Mitochondrial fission and fusion: molecular mechanisms, biological functions, and related disorders. Membranes.

[11] Schon K.R., Ratnaike T., van den Ameele J., Horvath R., Chinnery P.F. (2020). Mitochondrial diseases: a diagnostic revolution. Trends Genet.

[12] Stenton S.L., Prokisch H. (2020). Genetics of mitochondrial diseases: identifying mutations to help diagnosis. EBioMedicine.

[13] Nunnari J., Suomalainen A. (2012). Mitochondria: in sickness and in health. Cell.

[14] Calvo S.E., Mootha V.K. (2010). The mitochondrial proteome and human disease. Annu Rev Genom Hum Genet.

[15] Davis R.L., Liang C., Sue C.M. (2018). Mitochondrial diseases. Handb Clin Neurol.

[16] Lehtonen J.M., Auranen M., Darin N., Sofou K., Bindoff L., Hikmat O (2021). Diagnostic value of serum biomarkers FGF21 and GDF15 compared to muscle sample in mitochondrial disease. J Inherit Metab Dis.

[17] Montero R., Yubero D., Villarroya J., Henares D., Jou C., Rodríguez M.A. (2016). GDF-15 is elevated in children with mitochondrial diseases and is induced by mitochondrial dysfunction. PLoS One.

[18] Maresca A., Del Dotto V., Romagnoli M., La Morgia C., Di Vito L., Capristo M., ER-MITO Study Group (2020). Expanding and validating the biomarkers for mitochondrial diseases. J Mol Med (Berl).

[19] Stewart J.B. (2021). Current progress with mammalian models of mitochondrial DNA disease. J Inherit Metab Dis.

[20] Dunn D.A., Cannon M.V., Irwin M.H., Pinkert C.A. (2012). Animal models of human mitochondrial DNA mutations. Biochim Biophys Acta.

[21] Takahashi K., Tanabe K., Ohnuki M., Narita M., Ichisaka T., Tomoda K. (2007). Induction of pluripotent stem cells from adult human fibroblasts by defined factors. Cell.

[22] Fell C.W., Nagy V. (2021). Cellular models and high-throughput screening for genetic causality of intellectual disability. Trends Mol Med.

[23] Pang Z.P., Yang N., Vierbuchen T., Ostermeier A., Fuentes D.R., Yang T.Q. (2011). Induction of human neuronal cells by defined transcription factors. Nature.

[24] Tanabe K., Ang C.E., Chanda S., Olmos V.H., Haag D., Levinson D.F. (2018). Transdifferentiation of human adult peripheral blood T cells into neurons. Proc Natl Acad Sci U S A.

[25] McCombie W.R., McPherson J.D., Mardis E.R. (2019). Next-generation sequencing technologies. Cold Spring Harb Perspect Med.

[26] Margulies M., Egholm M., Altman W.E., Attiya S., Bader J.S., Bemben L.A. (2005). Genome sequencing in microfabricated high-density picolitre reactors. Nature.

[27] van Dijk E.L., Jaszczyszyn Y., Naquin D., Thermes C. (2018). The third revolution in sequencing technology. Trends Genet.

[28] Tankard R.M., Bennett M.F., Degorski P., Delatycki M.B., Lockhart P.J., Bahlo M. (2018). Detecting expansions of tandem repeats in cohorts sequenced with short-read sequencing data. Am J Hum Genet.

[29] Cao Y., Tokita M.J., Chen E.S., Ghosh R., Chen T., Feng Y. (2019). A clinical survey of mosaic single nucleotide variants in disease-causing genes detected by exome sequencing. Genome Med.

[30] Gambin T., Akdemir Z.C., Yuan B., Gu S., Chiang T., Carvalho C.M.B. (2017). Homozygous and hemizygous CNV detection from exome sequencing data in a Mendelian disease cohort. Nucleic Acids Res.

[31] Fromer M., Moran J.L., Chambert K., Banks E., Bergen S.E., Ruderfer D.M. (2012). Discovery and statistical genotyping of copy-number variation from whole-exome sequencing depth. Am J Hum Genet.

[32] Teer J.K., Mullikin J.C. (2010). Exome sequencing: the sweet spot before whole genomes. Hum Mol Genet.

[33] Warr A., Robert C., Hume D., Archibald A., Deeb N., Watson M. (2015). Exome sequencing: current and future perspectives. G3 Genes|Genomes|Genetics.

[34] French J.D., Edwards S.L. (2020). The role of noncoding variants in heritable disease. Trends Genet.

[35] Zhang F., Lupski J.R. (2015). Non-coding genetic variants in human disease. Hum Mol Genet.

[36] Logsdon G.A., Vollger M.R., Eichler E.E. (2020). Long-read human genome sequencing and its applications. Nat Rev Genet.

[37] Sanchis-Juan A., Megy K., Stephens J., Armirola Ricaurte C., Dewhurst E., Low K. (2023). Genome sequencing and comprehensive rare-variant analysis of 465 families with neurodevelopmental disorders. Am J Hum Genet.

[38] Nurk S., Koren S., Rhie A., Rautiainen M., Bzikadze A.V., Mikheenko A. (2022). The complete sequence of a human genome. Science.

[39] Liao W.-W., Asri M., Ebler J., Doerr D., Haukness M., Hickey G. (2023). A draft human pangenome reference. Nature.

[40] Wenger A.M., Peluso P., Rowell W.J., Chang P.-C., Hall R.J., Concepcion G.T. (2019). Accurate circular consensus long-read sequencing improves variant detection and assembly of a human genome. Nat Biotechnol.

[41] Wojcik M.H., Reuter C.M., Marwaha S., Mahmoud M., Duyzend M.H., Barseghyan H. (2023). Beyond the exome: what’s next in diagnostic testing for Mendelian conditions. Am J Hum Genet.

[42] Macken W.L., Vandrovcova J., Hanna M.G., Pitceathly R.D.S. (2021). Applying genomic and transcriptomic advances to mitochondrial medicine. Nat Rev Neurol.

[43] Lareau C.A., Dubois S.M., Buquicchio F.A., Hsieh Y.-H., Garg K., Kautz P. (2023). Single-cell multi-omics of mitochondrial DNA disorders reveals dynamics of purifying selection across human immune cells. Nat Genet.

[44] Walker M.A., Lareau C.A., Ludwig L.S., Karaa A., Sankaran V.G., Regev A. (2020). Purifying selection against pathogenic mitochondrial DNA in human T cells. N Engl J Med.

[45] Karczewski K.J., Francioli L.C., Tiao G., Cummings B.B., Alföldi J., Wang Q. (2020). The mutational constraint spectrum quantified from variation in 141,456 humans. Nature.

[46] Gudmundsson S., Singer-Berk M., Watts N.A., Phu W., Goodrich J.K., Solomonson M. (2022). Variant interpretation using population databases: lessons from gnomAD. Hum Mutat.

[47] Sirugo G., Williams S.M., Tishkoff S.A. (2019). The missing diversity in human genetic studies. Cell.

[48] Landrum M.J., Lee J.M., Riley G.R., Jang W., Rubinstein W.S., Church D.M. (2014). ClinVar: public archive of relationships among sequence variation and human phenotype. Nucleic Acids Res.

[49] Fokkema I.F.A.C., Taschner P.E.M., Schaafsma G.C.P., Celli J., Laros J.F.J., den Dunnen J.T. (2011). LOVD v.2.0: the next generation in gene variant databases. Hum Mutat.

[50] Stenson P.D., Mort M., Ball E.V., Chapman M., Evans K., Azevedo L. (2020). The Human Gene Mutation Database (HGMD®): optimizing its use in a clinical diagnostic or research setting. Hum Genet.

[51] Lott M.T., Leipzig J.N., Derbeneva O., Xie H.M., Chalkia D., Sarmady M. (2013). mtDNA variation and analysis using Mitomap and Mitomaster. Curr Protoc Bioinformatics.

[52] Richards S., Aziz N., Bale S., Bick D., Das S., Gastier-Foster J. (2015). Standards and guidelines for the interpretation of sequence variants: a joint consensus recommendation of the American College of medical genetics and genomics and the association for molecular pathology. Genet Med.

[53] Rentzsch P., Witten D., Cooper G.M., Shendure J., Kircher M. (2019). CADD: predicting the deleteriousness of variants throughout the human genome. Nucleic Acids Res.

[54] Ioannidis N.M., Rothstein J.H., Pejaver V., Middha S., McDonnell S.K., Baheti S. (2016). REVEL: an ensemble method for predicting the pathogenicity of rare missense variants. Am J Hum Genet.

[55] Cheng J., Novati G., Pan J., Bycroft C., Žemgulytė A., Applebaum T. (2023). Accurate proteome-wide missense variant effect prediction with AlphaMissense. Science.

[56] Brandes N., Goldman G., Wang C.H., Ye C.J., Ntranos V. (2023). Genome-wide prediction of disease variant effects with a deep protein language model. Nat Genet.

[57] Gao H., Hamp T., Ede J., Schraiber J.G., McRae J., Singer-Berk M. (2023). The landscape of tolerated genetic variation in humans and primates. Science.

[58] Frazer J., Notin P., Dias M., Gomez A., Min J.K., Brock K. (2021). Disease variant prediction with deep generative models of evolutionary data. Nature.

[59] Jaganathan K., Kyriazopoulou Panagiotopoulou S., McRae J.F., Darbandi S.F., Knowles D., Li Y.I. (2019). Predicting splicing from primary sequence with deep learning. Cell.

[60] T Z., Yi L. (2022). Predicting RNA splicing from DNA sequence using Pangolin. Genome Biol.

[61] Danzi M.C., Dohrn M.F., Fazal S., Beijer D., Rebelo A.P., Cintra V. (2023). Deep structured learning for variant prioritization in Mendelian diseases. Nat Commun.

[62] Tenney A.P., Di Gioia S.A., Webb B.D., Chan W.-M., de Boer E., Garnai S.J. (2023). Noncoding variants alter GATA2 expression in rhombomere 4 motor neurons and cause dominant hereditary congenital facial paresis. Nat Genet.

[63] Cheng J., Nguyen T.Y.D., Cygan K.J., Çelik M.H., Fairbrother W.G., Avsec Ž. (2019). MMSplice: modular modeling improves the predictions of genetic variant effects on splicing. Genome Biol.

[64] Scott, H.A., Place, E.M., Harper, E., Mehrotra, S., Cmg, B., Huckfeldt, R., Comander, J., Pierce, E.A., and Bujakowska, K.M. (2023). A high throughput splicing assay to investigate the effect of variants of unknown significance on exon inclusion. Preprint at medRxiv, 10.1101/2022.11.30.22282952. 10.1101/2022.11.30.22282952.

[65] Murdock D.R., Dai H., Burrage L.C., Rosenfeld J.A., Ketkar S., Müller M.F. (2021). Transcriptome-directed analysis for Mendelian disease diagnosis overcomes limitations of conventional genomic testing. J Clin Invest.

[66] Cummings B.B., Marshall J.L., Tukiainen T., Lek M., Donkervoort S., Foley A.R. (2017). Improving genetic diagnosis in Mendelian disease with transcriptome sequencing. Sci Transl Med.

[67] Gonorazky H.D., Naumenko S., Ramani A.K., Nelakuditi V., Mashouri P., Wang P. (2019). Expanding the boundaries of RNA sequencing as a diagnostic tool for rare mendelian disease. Am J Hum Genet.

[68] Stark R., Grzelak M., Hadfield J. (2019). RNA sequencing: the teenage years. Nat Rev Genet.

[69] Mullin N.K., Voigt A.P., Flamme-Wiese M.J., Liu X., Riker M.J., Varzavand K. (2023). Multimodal single-cell analysis of nonrandom heteroplasmy distribution in human retinal mitochondrial disease. JCI Insight.

[70] Wahedi A., Soondram C., Murphy A.E., Skene N., Rahman S. (2023). Transcriptomic analyses reveal neuronal specificity of Leigh syndrome associated genes. J Inherit Metab Dis.

[71] Frésard L., Smail C., Ferraro N.M., Teran N.A., Li X., Smith K.S. (2019). Identification of rare-disease genes using blood transcriptome sequencing and large control cohorts. Nat Med.

[72] Kremer L.S., Bader D.M., Mertes C., Kopajtich R., Pichler G., Iuso A. (2017). Genetic diagnosis of Mendelian disorders via RNA sequencing. Nat Commun.

[73] Yépez V.A., Gusic M., Kopajtich R., Mertes C., Smith N.H., Alston C.L. (2022). Clinical implementation of RNA sequencing for Mendelian disease diagnostics. Genome Med.

[74] Amarasinghe S.L., Su S., Dong X., Zappia L., Ritchie M.E., Gouil Q. (2020). Opportunities and challenges in long-read sequencing data analysis. Genome Biol.

[75] Bludau I., Aebersold R. (2020). Proteomic and interactomic insights into the molecular basis of cell functional diversity. Nat Rev Mol Cell Biol.

[76] Shuken S.R. (2023). An introduction to mass spectrometry-based proteomics. J Proteome Res.

[77] Cui M., Cheng C., Zhang L. (2022). High-throughput proteomics: a methodological mini-review. Lab Invest.

[78] Bennett H.M., Stephenson W., Rose C.M., Darmanis S. (2023). Single-cell proteomics enabled by next-generation sequencing or mass spectrometry. Nat Methods.

[79] Alfaro J.A., Bohländer P., Dai M., Filius M., Howard C.J., van Kooten X.F. (2021). The emerging landscape of single-molecule protein sequencing technologies. Nat Methods.

[80] Miller M.J., Kennedy A.D., Eckhart A.D., Burrage L.C., Wulff J.E., Miller L.A.D. (2015). Untargeted metabolomic analysis for the clinical screening of inborn errors of metabolism. J Inherit Metab Dis.

[81] Kennedy A.D., Wittmann B.M., Evans A.M., Miller L.A.D., Toal D.R., Lonergan S. (2018). Metabolomics in the clinic: a review of the shared and unique features of untargeted metabolomics for clinical research and clinical testing. J Mass Spectrom.

[82] Emwas A.-H.M. (2015). The strengths and weaknesses of NMR spectroscopy and mass spectrometry with particular focus on metabolomics research. Methods Mol Biol.

[83] Gertsman I., Barshop B.A. (2018). Promises and pitfalls of untargeted metabolomics. J Inherit Metab Dis.

[84] Mussap M., Zaffanello M., Fanos V. (2018). Metabolomics: a challenge for detecting and monitoring inborn errors of metabolism. Ann Transl Med.

[85] Liu N., Xiao J., Gijavanekar C., Pappan K.L., Glinton K.E., Shayota B.J. (2021). Comparison of untargeted metabolomic profiling vs traditional metabolic screening to identify inborn errors of metabolism. JAMA Netw Open.

[86] Manor J., Calame D.G., Gijavanekar C., Tran A., Fatih J.M., Lalani S.R. (2022). Niacin therapy improves outcome and normalizes metabolic abnormalities in an NAXD-deficient patient. Brain.

[87] Manor J., Calame D., Gijavanekar C., Fisher K., Hunter J., Mizerik E. (2022). NAXE deficiency: a neurometabolic disorder of NAD(P)HX repair amenable for metabolic correction. Mol Genet Metabol.

[88] Findlay G.M., Daza R.M., Martin B., Zhang M.D., Leith A.P., Gasperini M. (2018). Accurate classification of BRCA1 variants with saturation genome editing. Nature.

[89] Fowler D.M., Adams D.J., Gloyn A.L., Hahn W.C., Marks D.S., Muffley L.A. (2023). An Atlas of Variant Effects to understand the genome at nucleotide resolution. Genome Biol.

[90] Radford E.J., Tan H.K., Andersson M.H.L., Stephenson J.D., Gardner E.J., Ironfield H. (2022). Saturation genome editing of DDX3X clarifies pathogenicity of germline and somatic variation. Preprint at medRxiv.

[91] Ma K., Ng K., Huang S., Lake N., Xu J., Ge L. (2023). Using Saturation Mutagenesis-Reinforced Functional Assays (SMuRF) to improve the variant interpretation for alpha-dystroglycan glycosylation enzymes. Preprint at bioRxiv.

[92] Ebrahimi-Fakhari D., Alecu J.E., Brechmann B., Ziegler M., Eberhardt K., Jumo H. (2021). High-throughput imaging of ATG9A distribution as a diagnostic functional assay for adaptor protein complex 4-associated hereditary spastic paraplegia. Brain Communications.

[93] Ramezani M., Bauman J., Singh A., Weisbart E., Yong J., Lozada M. (2023). A genome-wide atlas of human cell morphology. Preprint at bioRxiv.

[94] Wang T., Birsoy K., Hughes N.W., Krupczak K.M., Post Y., Wei J.J. (2015). Identification and characterization of essential genes in the human genome. Science.

[95] Russell O.M., Gorman G.S., Lightowlers R.N., Turnbull D.M. (2020). Mitochondrial diseases: hope for the future. Cell.

[96] Cho S.-I., Lee S., Mok Y.G., Lim K., Lee J., Lee J.M. (2022). Targeted A-to-G base editing in human mitochondrial DNA with programmable deaminases. Cell.

[97] Lappalainen T., MacArthur D.G. (2021). From variant to function in human disease genetics. Science.

[98] Duan R., Hijazi H., Gulec E.Y., Eker H.K., Costa S.R., Sahin Y. (2022). Developmental genomics of limb malformations: allelic series in association with gene dosage effects contribute to the clinical variability. HGG Adv.

[99] Husain R.A., Grimmel M., Wagner M., Hennings J.C., Marx C., Feichtinger R.G. (2020). Bi-Allelic HPDL variants cause a neurodegenerative disease ranging from neonatal encephalopathy to adolescent-onset spastic paraplegia. Am J Hum Genet.

[100] Ghosh S.G., Lee S., Fabunan R., Chai G., Zaki M.S., Abdel-Salam G. (2021). Biallelic variants in HPDL, encoding 4-hydroxyphenylpyruvate dioxygenase-like protein, lead to an infantile neurodegenerative condition. Genet Med.

[101] Wiessner M., Maroofian R., Ni M.-Y., Pedroni A., Müller J.S., Stucka R. (2021). Biallelic variants in HPDL cause pure and complicated hereditary spastic paraplegia. Brain.

[102] Banh R.S., Kim E.S., Spillier Q., Biancur D.E., Yamamoto K., Sohn A.S.W. (2021). The polar oxy-metabolome reveals the 4-hydroxymandelate CoQ10 synthesis pathway. Nature.

[103] Van Bergen N.J., Guo Y., Rankin J., Paczia N., Becker-Kettern J., Kremer L.S. (2019). NAD(P)HX dehydratase (NAXD) deficiency: a novel neurodegenerative disorder exacerbated by febrile illnesses. Brain.

[104] Kremer L.S., Danhauser K., Herebian D., Petkovic Ramadža D., Piekutowska-Abramczuk D., Seibt A. (2016). NAXE mutations disrupt the cellular NAD(P)HX repair system and cause a lethal neurometabolic disorder of early childhood. Am J Hum Genet.

[105] Spiegel R., Shaag A., Shalev S., Elpeleg O. (2016). Homozygous mutation in the APOA1BP is associated with a lethal infantile leukoencephalopathy. Neurogenetics.

[106] Frost F.G., Morimoto M., Sharma P., Ruaud L., Belnap N., Calame D.G. (2023). Bi-allelic SNAPC4 variants dysregulate global alternative splicing and lead to neuroregression and progressive spastic paraparesis. Am J Hum Genet.

[107] Basel-Vanagaite L., Muncher L., Straussberg R., Pasmanik-Chor M., Yahav M., Rainshtein L. (2006). Mutated nup62 causes autosomal recessive infantile bilateral striatal necrosis. Ann Neurol.

[108] Lenk G.M., Szymanska K., Debska-Vielhaber G., Rydzanicz M., Walczak A., Bekiesinska-Figatowska M. (2016). Biallelic mutations of VAC14 in pediatric-onset neurological disease. Am J Hum Genet.

[109] Livingston J.H., Lin J.-P., Dale R.C., Gill D., Brogan P., Munnich A. (2014). A type I interferon signature identifies bilateral striatal necrosis due to mutations in ADAR1. J Med Genet.

[110] Peterson R.E., Kuchenbaecker K., Walters R.K., Chen C.-Y., Popejoy A.B., Periyasamy S. (2019). Genome-wide association studies in ancestrally diverse populations: opportunities, methods, pitfalls, and recommendations. Cell.

[111] Calame D.G., Guo T., Wang C., Garrett L., Jolly A., Dawood M. (2023). Monoallelic variation in DHX9, the gene encoding the DExH-box helicase DHX9, underlies neurodevelopment disorders and Charcot-Marie-Tooth disease. Am J Hum Genet.

[112] Calame D.G., Bakhtiari S., Logan R., Coban-Akdemir Z., Du H., Mitani T. (2021). Biallelic loss-of-function variants in the splicing regulator NSRP1 cause a severe neurodevelopmental disorder with spastic cerebral palsy and epilepsy. Genet Med.

[113] Calame D.G., Herman I., Maroofian R., Marshall A.E., Donis K.C., Fatih J.M. (2022). Biallelic variants in the Ectonucleotidase ENTPD1 cause a complex neurodevelopmental disorder with intellectual disability, distinct white matter abnormalities, and spastic paraplegia. Ann Neurol.

[114] Wright C.F., Campbell P., Eberhardt R.Y., Aitken S., Perrett D., Brent S. (2023). Genomic diagnosis of rare pediatric disease in the United Kingdom and Ireland. N Engl J Med.

[115] Wohler E., Martin R. (2021). GeneMatcher and VariantMatcher, tools for analysis and sharing of sequence data. Orphanet J Rare Dis.

[116] Calame D.G., Moreno Vadillo C., Berger S., Lotze T., Shinawi M., Poupak J. (2023). Cation leak through the ATP1A3 pump causes spasticity and intellectual disability. Brain.

[117] Paul M.S., Duncan A.R., Genetti C.A., Pan H., Jackson A., Grant P.E. (2023). Rare EIF4A2 variants are associated with a neurodevelopmental disorder characterized by intellectual disability, hypotonia, and epilepsy. Am J Hum Genet.

[118] Qian X., DeGennaro E.M., Talukdar M., Akula S.K., Lai A., Shao D.D. (2022). Loss of non-motor kinesin KIF26A causes congenital brain malformations via dysregulated neuronal migration and axonal growth as well as apoptosis. Dev Cell.

[119] Marafi D., Kozar N., Duan R., Bradley S., Yokochi K., Al Mutairi F. (2022). A reverse genetics and genomics approach to gene paralog function and disease: myokymia and the juxtaparanode. Am J Hum Genet.

[120] Mao D., Reuter C.M., Ruzhnikov M.R.Z., Beck A.E., Farrow E.G., Emrick L.T. (2020). De novo EIF2AK1 and EIF2AK2 variants are associated with developmental delay, leukoencephalopathy, and neurologic decompensation. Am J Hum Genet.

[121] Meng L., Isohanni P., Shao Y., Graham B.H., Hickey S.E., Brooks S. (2021). MED27 variants cause developmental delay, dystonia, and cerebellar hypoplasia. Ann Neurol.

[122] Bogaert E., Garde A., Gautier T., Rooney K., Duffourd Y., LeBlanc P. (2023). SRSF1 haploinsufficiency is responsible for a syndromic developmental disorder associated with intellectual disability. Am J Hum Genet.

[123] Faqeih E.A., Alghamdi M.A., Almahroos M.A., Alharby E., Almuntashri M., Alshangiti A.M. (2023). Biallelic variants in HECT E3 paralogs, HECTD4 and UBE3C, encoding ubiquitin ligases cause neurodevelopmental disorders that overlap with Angelman syndrome. Genet Med.

[124] Calame D.G., Hainlen M., Takacs D., Ferrante L., Pence K., Emrick L.T. (2020). EIF2AK2-related neurodevelopmental disorder with leukoencephalopathy, developmental delay, and episodic neurologic regression mimics pelizaeus-merzbacher disease. Neurol Genet.

[125] Dong X., Tan N.B., Howell K.B., Barresi S., Freeman J.L., Vecchio D. (2020). Bi-Allelic LoF NRROS variants impairing active TGF-β1 delivery cause a severe infantile-onset neurodegenerative condition with intracranial calcification. Am J Hum Genet.

[126] Maroofian R., Zamani M., Kaiyrzhanov R., Liebmann L., Ghayoor Karimiani E., Vona B. (2023). Biallelic variants in SLC4A10 encoding the sodium-dependent chloride-bicarbonate exchanger NCBE lead to a neurodevelopmental disorder. Genet Med.

[127] Caron V., Chassaing N., Ragge N., Boschann F., Ngu A.M.-H., Meloche E. (2023). Clinical and functional heterogeneity associated with the disruption of retinoic acid receptor beta. Genet Med.

[128] Duan R., Marafi D., Xia Z.-J., Ng B.G., Maroofian R., Sumya F.T. (2023). Biallelic missense variants in COG3 cause a congenital disorder of glycosylation with impairment of retrograde vesicular trafficking. J Inherit Metab Dis.

[129] Backman J.D., Li A.H., Marcketta A., Sun D., Mbatchou J., Kessler M.D. (2021). Exome sequencing and analysis of 454,787 UK Biobank participants. Nature.

[130] Denny J.C., Rutter J.L., Goldstein D.B., Philippakis A., Smoller J.W., Jenkins G., All of Us Research Program Investigators (2019). The “all of Us” research program. N Engl J Med.

[131] Davis R.L., Kumar K.R., Puttick C., Liang C., Ahmad K.E., Edema-Hildebrand F. (2022). Use of whole-genome sequencing for mitochondrial disease diagnosis. Neurology.

[132] Herman I., Jolly A., Du H., Dawood M., Abdel-Salam G.M.H., Marafi D. (2022). Quantitative dissection of multilocus pathogenic variation in an Egyptian infant with severe neurodevelopmental disorder resulting from multiple molecular diagnoses. Am J Med Genet.

[133] Owen M.J., Lefebvre S., Hansen C., Kunard C.M., Dimmock D.P., Smith L.D. (2022). An automated 13.5 hour system for scalable diagnosis and acute management guidance for genetic diseases. Nat Commun.

[134] Stenton S.L., O’Leary M., Lemire G., VanNoy G.E., DiTroia S., Ganesh V.S. (2023). Critical assessment of variant prioritization methods for rare disease diagnosis within the Rare Genomes Project. medRxiv.

[135] Chong J.X., Buckingham K.J., Jhangiani S.N., Boehm C., Sobreira N., Smith J.D. (2015). The genetic basis of mendelian phenotypes: discoveries, challenges, and opportunities. Am J Hum Genet.

[136] Smedley D., Smith K.R., Martin A., Thomas E.A., McDonagh E.M., Cipriani V., 100,000 Genomes Project Pilot Investigators (2021). 100,000 genomes pilot on rare-disease diagnosis in health care - preliminary report. N Engl J Med.

[137] Sun K.Y., Bai X., Chen S., Bao S., Kapoor M., Zhang C. (2023). A deep catalog of protein-coding variation in 985,830 individuals. bioRxiv.

[138] Pennisi E. (2022). Upstart DNA sequencers could be a “game changer.”. Science.

[139] Pais L.S., Snow H., Weisburd B., Zhang S., Baxter S.M., DiTroia S. (2022). seqr: a web-based analysis and collaboration tool for rare disease genomics. Hum Mutat.

[140] Soldatov V.O., Kubekina M.V., Skorkina M.Yu, Belykh A.E., Egorova T.V., Korokin M.V. (2022). Current advances in gene therapy of mitochondrial diseases. J Transl Med.

[141] Chernega T., Choi J., Salmena L., Andreazza A.C. (2022). Mitochondrion-targeted RNA therapies as a potential treatment strategy for mitochondrial diseases. Mol Ther Nucleic Acids.

[142] Salviati L., Trevisson E., Agosto C., Doimo M., Navas P., Adam M.P., Mirzaa G.M., Pagon R.A. (1993). GeneReviews®.

[143] Haack T.B., Klee D., Strom T.M., Mayatepek E., Meitinger T., Prokisch H. (2014). Infantile Leigh-like syndrome caused by SLC19A3 mutations is a treatable disease. Brain.

[144] Mayr J.A., Freisinger P., Schlachter K., Rolinski B., Zimmermann F.A., Scheffner T. (2011). Thiamine pyrophosphokinase deficiency in encephalopathic children with defects in the pyruvate oxidation pathway. Am J Hum Genet.

[145] Tabarki B., Al-Hashem A., Alfadhel M., Adam M.P., Mirzaa G.M., Pagon R.A. (1993). GeneReviews®.

[146] Gerards M., Kamps R., van Oevelen J., Boesten I., Jongen E., de Koning B. (2013). Exome sequencing reveals a novel Moroccan founder mutation in SLC19A3 as a new cause of early-childhood fatal Leigh syndrome. Brain.

[147] Pirinen E., Auranen M., Khan N.A., Brilhante V., Urho N., Pessia A. (2020). Niacin cures systemic NAD+ deficiency and improves muscle performance in adult-onset mitochondrial myopathy. Cell Metabol.

[148] Harel T., Yoon W.H., Garone C., Gu S., Coban-Akdemir Z., Eldomery M.K. (2016). Recurrent de novo and biallelic variation of ATAD3A, encoding a mitochondrial membrane protein, results in distinct neurological syndromes. Am J Hum Genet.

[149] Desai R., Frazier A.E., Durigon R., Patel H., Jones A.W., Dalla Rosa I. (2017). ATAD3 gene cluster deletions cause cerebellar dysfunction associated with altered mitochondrial DNA and cholesterol metabolism. Brain.

[150] Peeters-Scholte C.M.P.C.D., Adama van Scheltema P.N., Klumper F.J.C.M., Everwijn S.M.P., Koopmans M., Hoffer M.J.V. (2017). Genotype-phenotype correlation in ATAD3A deletions: not just of scientific relevance. Brain.

[151] Peralta S., González-Quintana A., Ybarra M., Delmiro A., Pérez-Pérez R., Docampo J. (2019). Novel ATAD3A recessive mutation associated to fatal cerebellar hypoplasia with multiorgan involvement and mitochondrial structural abnormalities. Mol Genet Metabol.

[152] Lepelley A., Della Mina E., Van Nieuwenhove E., Waumans L., Fraitag S., Rice G.I. (2021). Enhanced cGAS-STING-dependent interferon signaling associated with mutations in ATAD3A. J Exp Med.

[153] Dhir A., Dhir S., Borowski L.S., Jimenez L., Teitell M., Rötig A. (2018). Mitochondrial double-stranded RNA triggers antiviral signalling in humans. Nature.

[154] Green L., Hamilton N., Elpidorou M., Harris E.L., Douglas A., Ounap K. (2023). Biallelic mutation of SUPV3L1 causes an inherited leukodystrophy-associated neurodevelopmental disorder due to aberrant mitochondrial double stranded RNA processing. Preprint at medRxiv.

[155] van Esveld S.L., Rodenburg R.J., Al-Murshedi F., Al-Ajmi E., Al-Zuhaibi S., Huynen M.A. (2022). Mitochondrial RNA processing defect caused by a SUPV3L1 mutation in two siblings with a novel neurodegenerative syndrome. J Inherit Metab Dis.

[156] Crow Y.J., Stetson D.B. (2022). The type I interferonopathies: 10 years on. Nat Rev Immunol.

[157] Bamborschke D., Kreutzer M., Koy A., Koerber F., Lucas N., Huenseler C. (2021). PNPT1 mutations may cause Aicardi-Goutières-Syndrome. Brain Dev.

[158] Forny P., Footitt E., Davison J.E., Lam A., Woodward C.E., Batzios S. (2021). Diagnosing mitochondrial disorders remains challenging in the omics era. Neurology Genetics.

[159] Gunter C., Green E.D. (2023). To boldly go: unpacking the NHGRI's bold predictions for human genomics by 2030. Am J Hum Genet.

[160] Buergel T., Steinfeldt J., Ruyoga G., Pietzner M., Bizzarri D., Vojinovic D. (2022). Metabolomic profiles predict individual multidisease outcomes. Nat Med.

[161] Uddin M., Wang Y., Woodbury-Smith M. (2019). Artificial intelligence for precision medicine in neurodevelopmental disorders. npj Digit. Med..

[162] Foksinska A., Crowder C.M., Crouse A.B., Henrikson J., Byrd W.E., Rosenblatt G. (2022). The precision medicine process for treating rare disease using the artificial intelligence tool mediKanren. Front Artif Intell.

